# Potent Therapeutic Activity of NEO212 in Preclinical Models of Human and Canine Leukaemia and Lymphoma

**DOI:** 10.1111/vco.13066

**Published:** 2025-05-16

**Authors:** Thomas C. Chen, Steve Swenson, Thu Zan Thein, Radu O. Minea, Axel H. Schönthal

**Affiliations:** ^1^ Department of Neurosurgery, Keck School of Medicine University of Southern California (USC) Los Angeles California USA; ^2^ USC/Norris Comprehensive Cancer Center Los Angeles California USA; ^3^ NeOnc Technologies, Inc. Westlake Village California USA; ^4^ Department of Molecular Microbiology and Immunology Keck School of Medicine, USC Los Angeles California USA

**Keywords:** cancer therapy, DNA alkylation, O6‐methylguanine‐DNA methyltransferase, perillyl alcohol, temozolomide

## Abstract

Haematological cancer types, such as leukaemia and lymphoma, represent diseases that are life‐threatening to canine and human patients alike, and better treatments are needed. We are developing a novel anticancer agent, NEO212, a conjugate of two cancer drugs, the alkylating agent temozolomide (TMZ) and the monoterpene perillyl alcohol (POH). NEO212 has revealed robust therapeutic activity in preclinical tumour models harbouring different human cancer types. In the comparative preclinical study presented here, a two‐species (canine and human) and two‐cancer (leukaemia and lymphoma) analysis was performed to determine whether the promising therapeutic activity of NEO212 would span species and cancer types. We investigated the activity of NEO212 in human and canine leukaemia and lymphoma cell lines in vitro and in corresponding mouse models in vivo. Our results show that in vitro NEO212 is significantly more potent than TMZ and POH in all cell lines and exerts activity even against strongly TMZ‐resistant tumour cells. In vivo, oral NEO212 strikingly extends the survival of mice harbouring human or canine leukaemia or lymphoma cells. At the same time, NEO212 is well tolerated in dogs at dosages higher than those that achieved therapeutic activity in mouse models. Our study introduces NEO212 as a novel oral cancer drug candidate for both human and veterinary oncology applications.

**Abbreviations**
C‐3: caspase 3
C‐7: caspase 7
H2AX: H2A histone family member X
MGMT: O6‐methylguanine‐DNA methyltransferase
NEO100: pharmaceutical‐grade POH
NEO212: fusion product of TMZ and POH
O6BG: O6‐benzylguanine
p.o.: per oral
PARP‐1: poly (ADP‐ribose) polymerase 1
POH: perillyl alcohol
TMZ: temozolomide
WBC: white blood cells

## 1 Introduction

Comparative oncology, the study of cancer in multiple species, provides insights that may benefit several species [1]. We applied this approach to the study of NEO212, a novel molecule developed by NeOnc Technologies Inc. (Westlake Village, CA) as an anticancer therapeutic. It was generated by covalent conjugation of two previously characterised anticancer agents, perillyl alcohol (POH) and temozolomide (TMZ), and has recently entered Phase 1 clinical trials in patients with malignant brain cancers [clinicaltrials.gov ID NCT06047379] [2, 3, 4].

POH is a natural monoterpene related to limonene that is generated in plants through the mevalonate pathway and can be found, for example, in the essential oils of citrus fruit peel [5]. Preclinical studies established its anticancer activity in a large number of in vitro and in vivo tumour models [6, 7]. However, the clinical trials that followed were not successful, in part because the large doses of POH capsules that had to be ingested by cancer patients on a daily basis caused dose‐limiting gastrointestinal (GI) toxicities, and partly because its therapeutic activity was unconvincing [5, 7].

TMZ is an alkylating agent in clinical use for the treatment of malignant glioma. It is routinely included in the chemoradiation treatment protocol for these patients. Its therapeutic benefit, however, strongly depends on the O6‐methylguanine‐DNA methyltransferase (MGMT) status of the tumour tissue [8]. MGMT is a DNA repair protein able to remove the methyl moieties that are set by TMZ onto the O6‐position of guanine, thereby neutralising the key toxic impact of TMZ. As a consequence, patients with tumours that overexpress MGMT receive little to no benefit from TMZ therapy [9]. In addition, while TMZ is able to cross the blood–brain barrier (BBB) to reach brain‐localised malignancies, it does so rather inefficiently at a brain‐to‐plasma ratio of only 0.2 [10].

NEO212 emerged from an *in silico* search for new cancer drugs that might cross the BBB and enter the brain more efficiently [11]. Conjugation of POH and TMZ was predicted to fulfil this requirement, and we thus *de‐novo* synthesised NEO212. In a series of studies, we demonstrated several intriguing features of this new molecule that hold promise for the treatment of cancer. Compared with TMZ, NEO212 showed more potent anticancer activity in a variety of mouse tumour models, crossed the BBB more efficiently, and at the same time was better tolerated with less bone marrow toxicity in preclinical models. In addition, it revealed superior radio‐sensitising activity over TMZ when included in preclinical chemoradiation glioma models [2, 4, 12].

While NEO212 initially was being investigated as a novel brain cancer drug, we found that it harboured activity also against peripheral cancer types, such as melanoma, nasopharyngeal carcinoma, and lung cancer [13, 14, 15]. Its most striking therapeutic effect was observed in mouse models of acute myeloid leukaemia (AML) [16, 17], which prompted us to investigate NEO212's impact on other haematological cancer types as well. In the current report, we present the activity of NEO212 against human and canine leukaemia and lymphoma cells in vitro and in vivo. We demonstrate that this novel drug exerts benefit against cancer types from both species, and that healthy dogs tolerate NEO212 at doses that proved to be therapeutic in preclinical mouse tumour models.

## 2 Material and Methods

### 2.1 Pharmacological Agents

NEO212 was originally synthesised by Norac Pharma (Azusa, CA) and subsequently made to order by Axon MedChem (Reston, VA). It is an off‐white, crystalline powder that was kindly provided by NeOnc Technologies Inc. (Westlake Village, CA). It was dissolved in DMSO at 100 or 500 mM for in vitro or in vivo experiments, respectively. TMZ (Sigma Aldrich, St Louis, MO) was dissolved in DMSO to a concentration of 100 mM. 06BG (Santa Cruz Biotechnology, Santa Cruz, CA) was dissolved in DMSO to a concentration of 50 mM. Aliquots of these stock solutions were stored at −80°C. POH was obtained as NEO100, a highly pure version of POH that was produced under current good manufacturing practice (CGMP) conditions by Norac Pharma and provided by NeOnc Technologies. It is an oily liquid that was stored refrigerated at 4°C. It was diluted fresh before each use with DMSO to a 600 mM stock solution, then diluted further with medium. In all cases of cell treatment, the final DMSO concentration in the culture medium never exceeded 0.3% and was much lower in most cases.

### 2.2 Cells Lines

The representative canine cell lines were as follows: CLBL1 is a B‐cell lymphoma cell line established from a dog with stage IV diffuse large‐cell lymphoma [18]. These cells were provided by Barbara Rose in the laboratory of Douglas H. Thamm (Flint Animal Cancer Center, Colorado State University, Fort Collins, CO) with permission obtained from Barbara C. Rütgen (University of Veterinary Medicine, Vienna, Austria). CLL1390 cells represent primitive T‐cell leukaemia [19]. These cells were provided by Kristy Harmon in the laboratory of Peter F. Moore (UC Davis School of Veterinary Medicine, Davis, CA). Both canine cell lines were authenticated by IDEXX BioAnalytics (Columbia, MO); they were also confirmed to be negative for mycoplasma. The following human cell lines were obtained from the American Tissue Culture Collection (ATCC, Manassas, VA): The HL60 cell line was established from a patient with acute promyelocytic leukaemia [20]. THP1 cells were derived from an acute monocytic leukaemia patient [21]. U937 cells were established from a patient with histiocytic lymphoma and exhibit monocyte morphology [22]. Raji cells represent B lymphocytes and were established as the first continuous human cell line of haematopoietic origin from a patient with Burkitt's lymphoma [23]. These human cell lines were confirmed to be negative for mycoplasma, but were not authenticated due to use at low passage after receipt from ATCC (providing certified authenticated cells).

### 2.3 Cell Culture Conditions; Immunoblots; MTT Assays; Cell Death Analysis by FACS; Isolation of RNA and RT‐qPCR

See Supporting Information for details.

### 2.4 Animal Tumour Models

All mouse protocols were reviewed and approved by the Institutional Animal Care and Use Committee (IACUC) of the University of Southern California (USC). We obtained immune‐deficient, female 6–8‐week‐old NOD‐SCID or NSG‐SGM3 mice from the Jackson Laboratory (Bar Harbour, ME). The animals were housed at the USC Medical Center Animal Facility, which is AAALAC and AALAS certified and has written animal welfare assurance with the NIH Office of Laboratory Animal Welfare (OLAW) that commits the institution to follow the standards established by the Animal Welfare Act [24]. Details of tumour cell implantation, drug treatment, and criteria for euthanasia of these mice are provided in the Supporting Information.

### 2.5 Toxicology Studies

Toxicology studies in beagle dogs were performed at Charles River Laboratories (CRL; Montreal, Canada). All animal studies at CRL are subject to oversight by the IACUC, Ethics Committee (EC), and Animal Welfare Body (AWB) to ensure that all animal care and research meet or exceed the relevant local and national legislation and guidelines, including the *Guide for the Care and Use of Laboratory Animals* [25], published by the Institute for Laboratory Animal Resources (ILAR). Veterinary care was available throughout the study. The animals were 5 to 7 months old and weighed between 5.9 and 8.0 kg (males) and 5.1 and 7.0 kg (females). Animals were socially housed (up to 3 dogs per cage) and were acclimated to the oral procedure for at least 3 days prior to the commencement of dose formulation administration by using empty capsules.

### 2.6 Statistical Analysis

All parametric data were analysed using the Student's *t*‐test to calculate the significance values. For animal survival, a log‐rank (Mantel‐Cox) test was used. A probability value (*p*) < 0.05 was considered statistically significant.

## 3 Results

We conducted a two‐species, two‐disease investigation with the overall goal of introducing NEO212 into clinical and veterinary practice as a novel oncology drug. The following cell lines were used as models: canine CLBL1 lymphoma and CLL1390 leukaemia; human Raji lymphoma and HL60, THP1, and U937 leukaemia. The chemical structure of NEO212 is shown in Figure S1.

### 3.1 NEO212 is More Cytotoxic Than TMZ or NEO100

**FIGURE 1:**
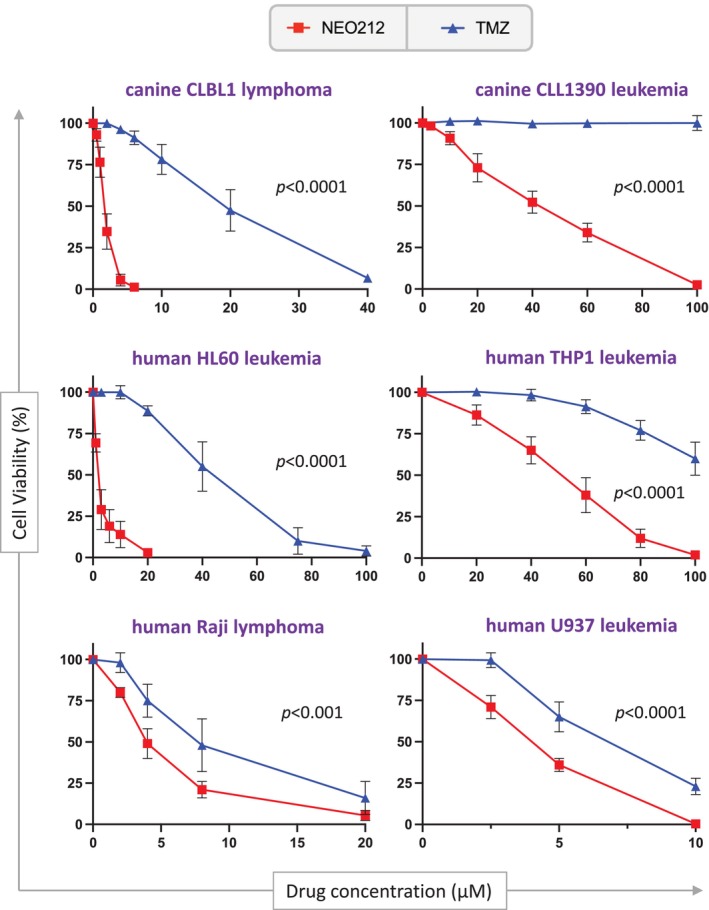
NEO212 reduces cell viability more than TMZ. Several human and canine leukaemia and lymphoma cell lines were exposed to increasing concentrations of NEO212 or TMZ, and cell viability was determined 4 days later by MTT assay. Shown are averages (*n* ≥ 3 ± SD), where the viability of vehicle‐treated cells was set to 100% (treatment with vehicle did not show any difference to untreated cells). The *p*‐values shown refer to a comparison of cell viability percentages at the same drug concentrations (*n* ≥ 3) used for NEO212 and TMZ, respectively, as shown in each panel.

Cells were treated with increasing concentrations of NEO212, and cell viability was determined with MTT assays. TMZ was used as a clinically relevant alkylating agent comparison. As shown in Figure 1, NEO212 reduced the survival of all cell lines in a dose‐dependent manner, and this effect was significantly (*p* < 0.001 and *p* < 0.0001) more potent than that of TMZ. Depending on the cell line, the IC50 of NEO212 ranged from 1.7 to about 50 μM (Table 1).

**TABLE 1:** Inhibitory concentrations of different drug treatments.

A: CLBL1	IC50 (range)^a^	IC90 (range)^a^
NEO212	1.7 (1.1–4.2)	3.8 (3.2–5.6)
TMZ	19 (16–29)	39 (32–51)
NEO100	382 (304–487)	739 (689–844)
TMZ + NEO100	19 (14–30)	39 (28–51)

B: CLL1390	IC50 (range)^a^	IC90 (range)^a^
NEO212	42 (34–49)	90 (76–103)
TMZ	> 200 (> 200)	> 200 (> 200)
NEO100	280 (251–341)	722 (581–860)
TMZ + NEO100	> 200 (> 200)	> 200 (> 200)

*Note*: Each value represents the average (range) value obtained from 3 to 4 independent repeats of MTT assays (set up in triplicate or quadruplicate). TMZ was not applied at concentrations above 200 μM, because the physiological range only reaches 75 μM.

^a^ Range in μM.

**FIGURE 2:**
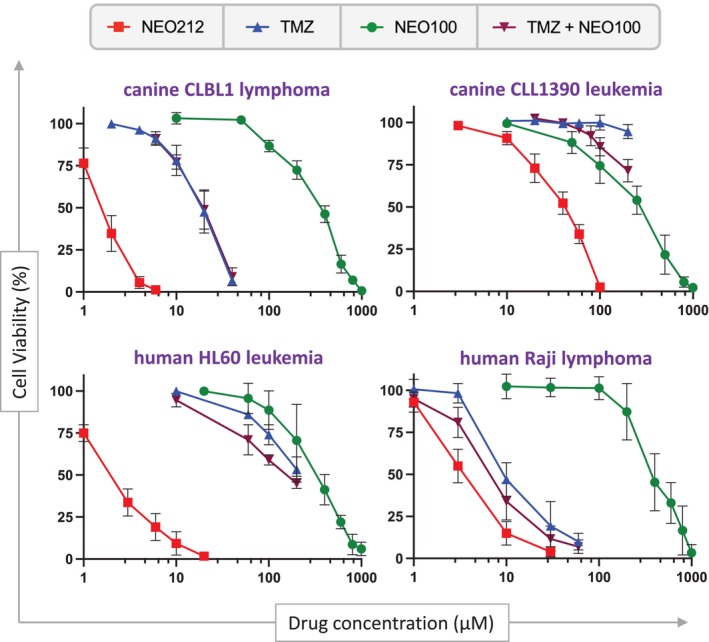
Greater in vitro potency of NEO212 cannot be mimicked by TMZ + POH/NEO100 combination treatment. Cells were treated with NEO212, TMZ, NEO100, or with TMZ mixed with NEO100 at equimolarconcentrations, and cell viability was determined 4 days later by MTT assay. Shown are averages (*n* ≥ 3 ± SD), where the viability of vehicle‐treated cells was set to 100%. *Equimolar is depicted on the *x*‐axis as 1 data point, for example, the data point at 100 μM resulted from the combination treatment of cells with 100 μM TMZ mixed with 100 μM NEO100. TMZ alone and in combination was only used at concentrations up to 200 μM, because the upper limit of TMZ concentrations reached in the plasma of cancer patients does not exceed 50–75 μM [27].

Because NEO212 is a hybrid compound composed of TMZ conjugated to POH, we next investigated whether an equimolar mix of TMZ and POH as individual agents would be able to mimic the strong potency of the conjugated molecule. In place of POH, we used NEO100, which is the pharmaceutical‐grade, highly pure version of POH that is used in currently ongoing clinical trials [26]. Cells were treated with NEO212, TMZ, NEO100, or TMZ combined with NEO100, and cell viability was again determined with MTT assays. As displayed in Figure 2, treating cells with an equimolar mix of TMZ plus NEO100 was not able to mimic the superior cytotoxic potency of NEO212. POH/NEO100 is known as a relatively weak anticancer agent, and consistent with this expectation showed a rather high IC50 of well above 100 μM; when added to TMZ, it did not significantly lower the IC50 that was observed with TMZ alone, that is, the combination treatment was not substantially more potent than TMZ by itself. This was particularly obvious for the two canine cell lines, where neither the IC50 nor the IC90 of TMZ monotherapy could be enhanced by the addition of NEO100 (Figure 2, Table 1). Clearly, the cytotoxic activity of the conjugated compound NEO212 was greater than the sum of its parts. (In these experiments, TMZ was only used at concentrations up to 200 μM, because the upper limit of TMZ concentrations reached in the plasma of cancer patients does not exceed 50–75 μM [27]. Therefore, much higher TMZ concentrations lack physiological relevance).

### 3.2 MGMT Expression Levels Inversely Correlate With NEO212 Sensitivity in Canine Cells

It was of interest that the IC50 values achieved by NEO212 varied by a rather large margin between the different cell lines. In our previous studies with human cancer cells, we showed that this effect was caused in large part by the presence of the MGMT DNA repair protein, which confers strong chemoresistance against the DNA‐alkylating impact of TMZ. We therefore investigated this aspect in the two canine cell lines CLBL1 and CLL1390, where NEO212's IC50 was 1.7 and 42 μM, respectively (Table 1), and the IC50 of TMZ was 19 and > 200 μM (the latter indicating profound TMZ resistance in the CLL1390 line).

**FIGURE 3:**
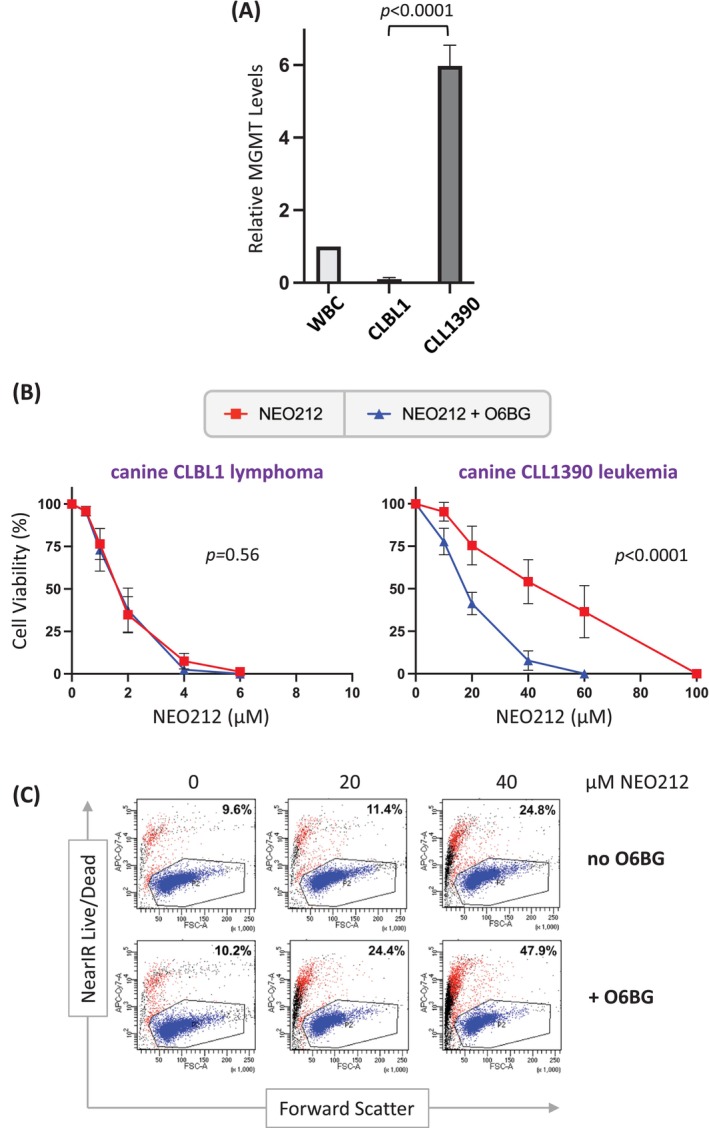
Canine cells have different expression levels of MGMT. (A) Relative MGMT mRNA levels were determined by RT‐qPCR as described under Methods. The very low signal obtained in CLBL1 cells represents the detection limit, that is, these cells can be considered MGMT negative. WBC: White blood cells obtained from a healthy dog. (B) Cells were treated with increasing concentrations of NEO212 in the presence or absence of 15 μM O6BG, and cell viability was determined 4 days later by MTT assay. (C) CLL1390 cells were treated with 0, 20, or 40 μM NEO212 in the presence or absence of 15 μM O6BG. Five days later, FACS was used to separate the cell populations into dead (red/black dots) or surviving (blue dots) fractions, using a LIVE/DEAD Near‐IR Dead cell kit from Thermo Fisher Scientific (see details in Supporting Information). The indicated percentages reflect the fractions of dead cells. Note that O6BG alone had no effect, but its inclusion in NEO212 treatment doubled the percentage of dead cells at the end of treatment.

First, MGMT expression in CLBL1 and CLL1390 cells was investigated by RT‐qPCR. As shown in Figure 3A, MGMT expression levels in CLBL1 cells were below the detection limit, whereas CLL1390 cells displayed high levels. As a comparison, we also analysed white blood cells derived from a blood draw from a healthy dog, which showed intermediate MGMT transcript levels. Second, we used O6BG, a well‐known inhibitor of MGMT protein function [28]. Cells were treated with NEO212 in the presence or absence of O6BG, followed by the MTT viability assay. We found that O6BG significantly lowered the IC50 of NEO212 (from 44 to 19 μM) in CLL1390 cells but had no such effect in CLBL1 cells (Figure 3B). Third, the inclusion of O6BG in the NEO212 treatment of CLL1390 cells significantly increased (i.e., doubled) the fraction of dead and dying cells, as analysed by FACS (Figure 3C). We also attempted Western blot analysis of MGMT protein as a direct readout. However, those results remained inconsistent. We tested different MGMT‐specific antibodies that readily reacted with human and mouse MGMT but apparently were unable to recognise the canine homologue. Nonetheless, our results obtained at the transcript level, in combination with the O6BG effects, strongly indicate that the higher IC50 value of CLL1390 cells against NEO212, at least in part, is due to the elevated expression of MGMT in these cells.

### 3.3 NEO212 Triggers Apoptosis

**FIGURE 4:**
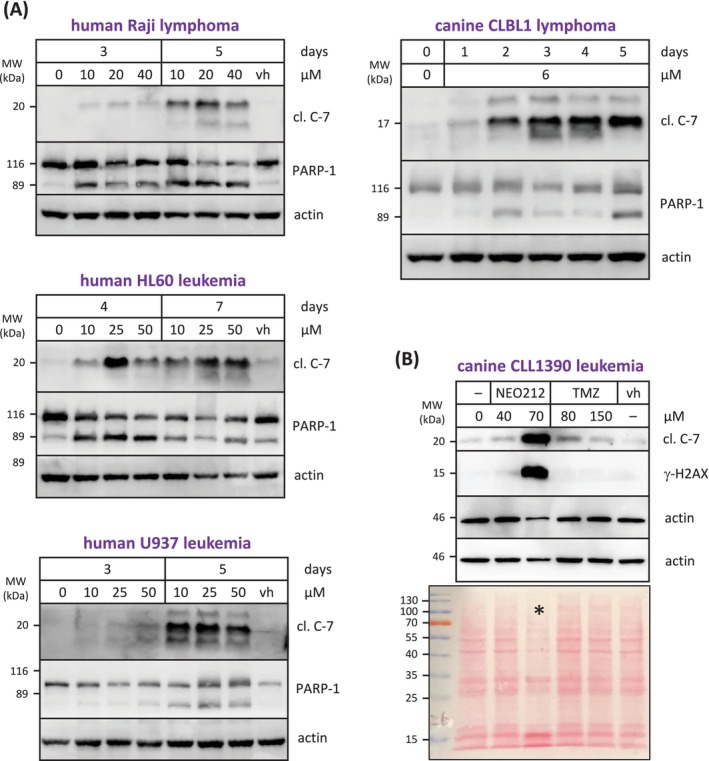
NEO212 triggers apoptosis. The induction of apoptotic cell death was determined by Western blot analysis with antibodies recognising typical markers of apoptosis: Cleaved caspase 7 (cl. C‐7) and PARP‐1, and phosphorylated H2AX (γ‐H2AX). Actin was used as the loading control. (A) Cells were treated with various concentrations of NEO212 for different times, as indicated. Control cells remained untreated (0) or received vehicle only (vh). (B) Cells were treated with NEO212, TMZ, or vehicle for 3 days. The actin result is shown from two different experiments; it consistently revealed a lower signal for this loading control at the higher concentration for NEO212. Staining of the membrane after gel transfer with Ponceau red (shown at the bottom) revealed the deterioration of cellular proteins at 70 μM NEO212 (lane marked with an asterisk), consistent with end‐stage apoptosis.

To gain insight into the underlying mechanisms of cell death, we next investigated three established markers of apoptosis: proteolytic cleavage of caspase 7 (C‐7) and poly (ADP‐ribose) polymerase 1 (PARP‐1), and phosphorylation of H2A histone family member X (γ‐H2AX). All three proteins were analysed by Western blot. As summarised in Figure 4, the treatment of cells with NEO212 resulted in the prominent appearance of these apoptosis markers. Of note, the onset of apoptosis was a slow process that became much more evident several days after the addition of drug to the cells. This delayed kinetic has been reported before for TMZ and is consistent with O6‐guanine‐DNA methylation as the key cytotoxic event conveyed by these drugs [8].

We further compared apoptosis between NEO212 and TMZ treatment in the TMZ‐resistant CLL1390 cells. As shown in Figure 4B, NEO212 was dramatically more potent in triggering the emergence of apoptosis markers than TMZ. While 70 μM NEO212 caused striking C‐7 cleavage and prominent phosphorylation of H2AX, TMZ at more than double this concentration did not show an apparent effect on these markers. Similarly, Ponceau red staining of electrophoresed cellular proteins revealed the onset of proteolytic deterioration of cellular contents in response to NEO212, but not after TMZ treatment (Figure 4B). These results were consistent with those obtained from MTT assays (Figure 1) and emphasised the robust activity of NEO212 even against highly TMZ‐resistant cells.

**FIGURE 5:**
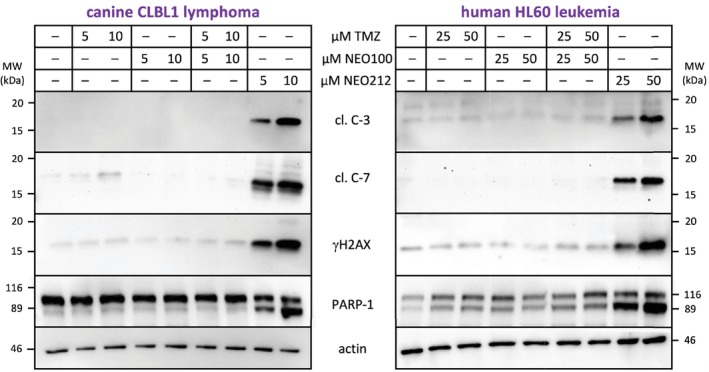
Combination treatment with individual components, TMZ + NEO100, does not trigger apoptosis. The induction of apoptotic cell death was determined by Western blot analysis with antibodies recognising typical markers of apoptosis, that is, proteolytic cleavage of caspase 3 (cl. C‐3), caspase 7 (cl. C‐7), and PARP‐1, and phosphorylation of H2AX (γ‐H2AX). Actin was used as the loading control. Cells were treated with two different concentrations each of TMZ or NEO100, either alone or in combination, or with the same two different concentrations of NEO212, as indicated in the figure. Control cells remained untreated. All cell populations were collected 72 h later.

As was shown in Figure 2 with MTT assays, an equimolar mix of TMZ plus NEO100 was unable to mimic the superior anticancer activity of NEO212. To further validate that finding, we next investigated the response of apoptosis markers to TMZ plus NEO100 combination treatment and compared it to the apoptotic response to NEO212, where the two agents are covalently conjugated. The results reveal that a mix of TMZ and NEO100 is unable to replicate the much stronger potency of NEO212 (Figure 5). For example, treating canine CLBL1 cells with a combination of 10 μM TMZ mixed with 10 μM NEO100 was unable to trigger the expression of any of the apoptotic proteins examined, whereas NEO212 as low as 5 μM caused prominent cleavage (i.e., activation) of caspase 3, caspase 7, and PARP‐1, and strong phosphorylation of H2AX protein. Similarly, in human HL60 cells, the combination of 50 μM TMZ and 50 μM NEO100 was unable to mimic the appearance of apoptotic markers that was triggered by NEO212 at concentrations as low as 25 μM. These results confirm that the potency of NEO212 is greater than the sum of its parts.

### 3.4 NEO212 is Active in Leukaemia and Lymphoma Models *In Vivo*

The in vivo anticancer activity of NEO212 was investigated in immune‐deficient mice implanted with human or canine leukaemia or lymphoma cells. In all, five models were used. Seven to twelve days after tumour cell seeding, mice were subjected to two cycles of drug treatment (or vehicle only) at 25 mg/kg NEO212 or 25 mg/kg TMZ per dose (one dose per day). Each cycle consisted of once‐daily oral gavage for five consecutive days, separated by a few days of a treatment holiday. Thereafter, mice were monitored for their health and behaviour in the absence of any further drug administration. The survival of all animals was recorded and graphed in Kaplan–Meier survival curves.

**FIGURE 6:**
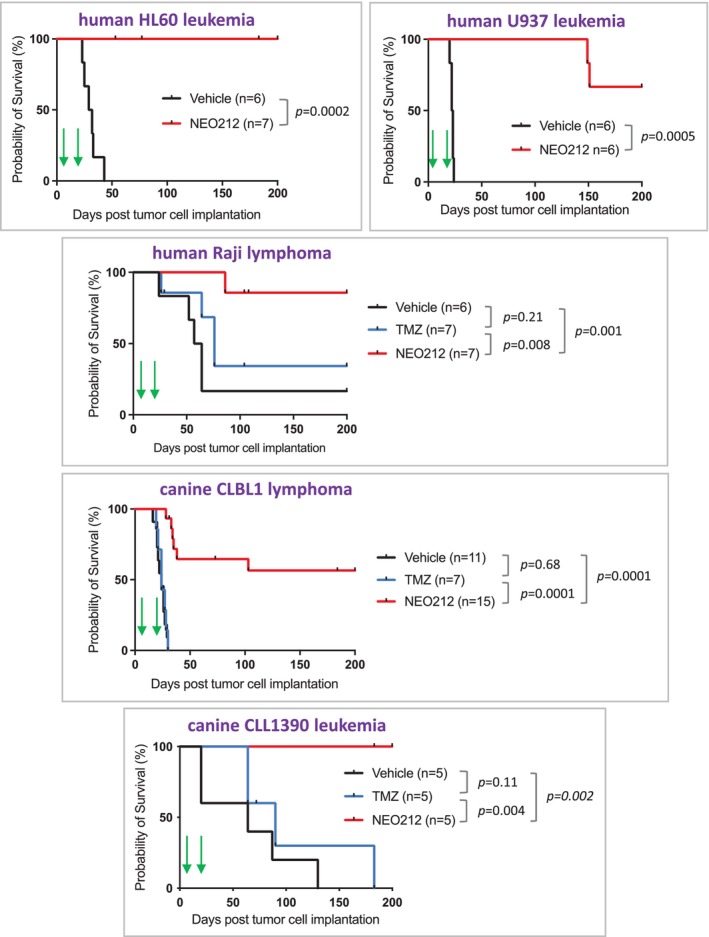
NEO212, but not TMZ, prolongs survival of mice with xenografted tumour cells. Mice implanted with leukaemia or lymphoma cells were treated with 25 mg/kg NEO212 or vehicle only. Groups of mice injected with Raji, CLBL1, or CLL1390 cells also received 25 mg/kg TMZ. In all cases, drugs were administered by daily oral gavage on a schedule of 5 days on—9 days off—5 days on, that is, two cycles total (marked by green arrows). Shown are Kaplan–Meier survival plots. Tick marks indicate censored data (e.g., when an animal had to be removed due to injuries from infighting unrelated to disease). Statistical comparisons between groups were performed with a log‐rank test.

As summarised in Figure 6, the treatment of tumour‐bearing mice with NEO212 significantly prolonged their survival, and this benefit was apparent in all 5 tumour models. Depending on the specific model used, the median survival of vehicle‐treated control mice ranged from 25 to 65 days. In comparison, NEO212‐treated mice survived much longer, and at the end of the experiment at 200 days, the majority of these animals were still alive. In fact, in the case of human HL60 leukaemia and canine CLL1390 leukaemia, no animals in the NEO212‐treated groups succumbed to disease within the 200‐day time frame.

In one of the human cancer models (Raji lymphoma) and in both of the canine cancer models, we also included TMZ as a treatment modality, given at the same dose and schedule as NEO212. In all three cases, however, TMZ was ineffective, that is, it did not significantly improve the survival of treated mice as compared to the vehicle‐treated control mice. In all, our in vivo experiments established potent in vivo anticancer activity of NEO212 in these leukaemia and lymphoma models, and when compared with TMZ, NEO212 proved superior.

### 3.5 NEO212 at 50 Mg/Kg/Day (×5) is Tolerated by Dogs

The safety of NEO212 as a therapeutic agent had been investigated in previous studies using rodents. Those studies showed that this novel compound was well tolerated and caused less severe side effects, such as myelosuppression, than TMZ [2, 13, 14, 16]. In preparation for clinical and veterinary applications, toxicology studies were performed in beagle dogs at Charles River Laboratories (CRL) under Good Laboratory Practice (GLP) conditions. The objective was to determine the potential toxicity of NEO212 when given daily by oral capsule for 5 days, and to evaluate the possible reversibility of any findings within a 23‐day recovery phase. This particular treatment schedule was chosen because it represents the envisioned therapeutic regimen in patients and is based on the established routine use of adjuvant TMZ in cancer patients.

**FIGURE 7:**
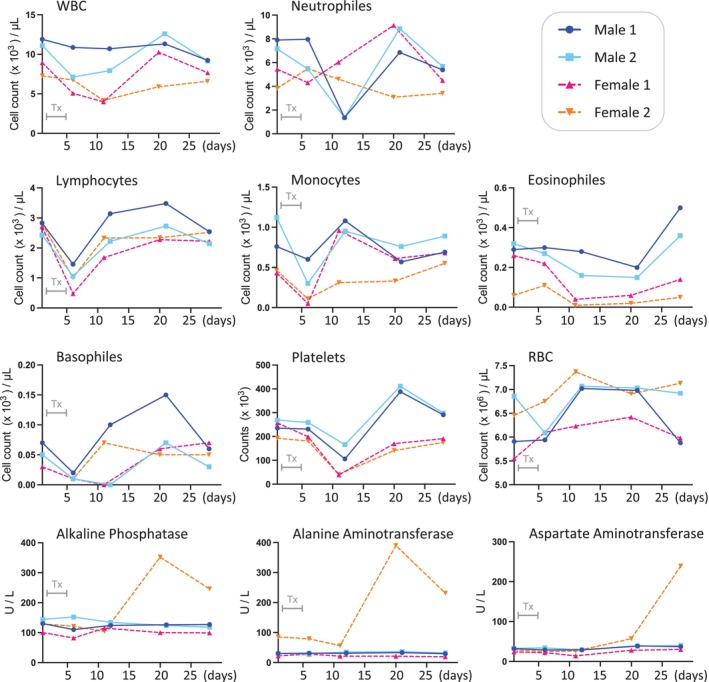
NEO212 is tolerated by dogs. Two male and two female beagle dogs were subjected to once‐daily oral pilling with 50 mg/kg NEO212 for 5 days. Blood draws were performed on each animal before the first dosing, at 6 days, at 11 (females) and 12 (male) days, at 20 (females) and 21 (males) days, and at 28 days. The blood samples were subjected to complete blood counts (CBC) with differential and measurements of key liver enzymes. Grey bar labelled “Tx” in each panel indicates the 5‐day drug treatment period.

Four dogs (2 males +2 females) received 50 mg/kg NEO212 p.o. once daily for 5 consecutive days. Blood draws were performed in each animal before the start of treatment and at regular intervals thereafter. Complete blood counts (CBC) with differential showed that NEO212 caused lower white blood cell (WBC) counts with associated subpopulations (Figure 7) and increased liver enzymes in one dog. The red blood cell (RBC) count was lower only in one dog, not in the other three. In all cases, however, lower cell counts eventually rebounded during the recovery phase. Several additional parameters, including food consumption and body weight, blood chemistry and liver values, urinalysis, ophthalmology, electrocardiology and others, were evaluated also. In all instances, no severe adverse events became apparent, and by the end of the month, all four dogs had fully recovered from treatment, indicating that a dose of 50 mg/kg was tolerated by these animals.

## 4 Discussion

Our study aimed to determine whether NEO212 exerts therapeutic potential in preclinical models of human and canine leukaemia and lymphoma, and whether dogs would tolerate a 5‐day cycle of repeated dosing. The current results show that NEO212 effectively triggered apoptotic cell death in all tested cell lines in vitro, and exerted potent anticancer activity in leukaemia/lymphoma models in vivo. Five‐day repeat dosing of NEO212 in beagle dogs was tolerated and did not cause severe adverse events. In all, these results bode well for the use of NEO212 in the clinical and veterinary setting, and our study suggests that this novel compound should be evaluated in larger cohorts of patients.

Prior studies investigating NEO212 have established its anticancer activity in a variety of preclinical models representing human cancer types [2, 4, 12, 13, 14, 15, 16, 17]. These studies consistently demonstrated that NEO212 proved superior not only to TMZ, but also to a mix of its subunits as individual compounds. The current study expands this promising effect to canine leukaemia and lymphoma. Both of these diseases present with different subtypes in canine patients, and just like their human counterparts, they can be life‐threatening and require carefully planned treatment regimens [29, 30]. Although leukaemia is rather uncommon in dogs, animals of any breed and at any age can develop this disease. Acute leukaemia is particularly aggressive and is almost exclusively treated with chemotherapy, including prednisone, cyclophosphamide, doxorubicin, and other drugs, but is rarely cured. In the case of lymphoma, similar chemotherapy options exist, many of which are given via intravenous infusion and usually require access to a specialty centre. An exception is verdinexor (Laverdia‐CA1, Dechra), the first oral treatment for dogs with lymphoma conditionally approved by the FDA [31]. In general, oral drugs provide several advantages over intravenous interventions, such as non‐invasiveness, reduced risk for cannula‐related infections, fewer clinic visits, greater patient compliance and convenience, and usually less cost. It is therefore noteworthy that NEO212 is being developed as an oral medication.

TMZ is an oral drug as well as part of the standard chemoradiation regimen applied to human malignant glioma patients [8]. However, its efficacy is severely reduced when tumour cells express the MGMT protein, and this limitation applies to other cancer types also. In human acute myeloid leukaemia (AML) patients, for example, TMZ demonstrated modest clinical activity, but only when patients were pre‐screened for low MGMT expression [32, 33]. Similarly, patients with primary central nervous system lymphoma (PCNSL) responded to TMZ better when their tumour cells harboured low MGMT levels [34]. In veterinary patients, TMZ has shown limited clinical activity so far [35, 36, 37, 38]. While MGMT represents an important component of tumour cell resistance against TMZ, other tumour‐inherent factors play critical roles as well, both in human and canine tumours [39, 40, 41]. In the in vitro part of our study, we observed that NEO212 was active against all cell lines, irrespective of their MGMT status, although higher drug concentrations were needed in MGMT‐expressing cells. For instance, in MGMT‐positive CLL1390 cells, the IC50 of NEO212 was 42 μM, whereas the IC50 of TMZ was well above 200 μM. As a reference, the upper limit of TMZ concentrations reached in the plasma of human cancer patients is in the range of 50–75 μM [27]. Furthermore, the addition of NEO100/POH to TMZ, that is, a combination treatment of cells with the individual compounds that make up NEO212, is unable to reach the superior anticancer activity of NEO212, consistent with our earlier findings in other preclinical human cancer models [2, 4, 13, 14, 15].

NEO212 revealed robust anticancer activity in vivo as well. In five mouse tumour models, three with human and two with canine cells, the survival of NEO212‐treated animals was strikingly prolonged as compared to vehicle‐treated mice. This outcome was particularly impressive given that only two 5‐day cycles of NEO212 were administered; there were no further treatments of any kind beyond Day 30. In stark contrast, treatment with TMZ in the same manner did not result in a survival advantage, further emphasising the superior therapeutic benefit of NEO212.

We further demonstrated that beagle dogs were able to tolerate a 5‐day course of 50 mg/kg/day NEO212 (equivalent to 850–990 mg/m^2^/day for dogs with a body weight of 5–8 kg, as used in our study). The main toxicities were haematological and, in one female dog, elevated liver enzymes. Intriguingly, an earlier phase 1 study using TMZ on the same treatment schedule in client‐owned dogs with a range of advanced cancers established the MTD of TMZ at 150 mg/m^2^/day (equivalent to 7.6–8.9 mg/kg for 5–8 kg dogs) [42]. Despite the much lower MTD as compared to our observations with NEO212, similar haematologic and hepatic adverse events were noted in that dose‐finding study. This very large differential in tolerated doses between NEO212 and TMZ was also documented in a toxicity study in rats: It was reported that a 5‐day course of daily 200 mg/kg TMZ resulted in severe myelosuppression and subsequent death of all (*n* = 3) treated animals, whereas the same dose and schedule of NEO212 caused only a mild, reversible decline in WBC counts with full recovery of the animals (*n* = 3) [16]. Combined, these data need to be viewed in the context of in vivo efficacy studies, where the side‐by‐side comparison of NEO212 with TMZ consistently showed that NEO212 achieved superior therapeutic outcomes when administered at the same dose [2, 4, 13, 15]. One can therefore conclude that NEO212 is not only strikingly more effective, but also significantly better tolerated than TMZ.

In summary, our results bode well for the further development of NEO212 as a cancer therapeutic for veterinary oncology. While this novel agent has entered a phase 1 clinical trial to evaluate its safety in human brain cancer patients, we are proposing that it should also be investigated for its potential benefit for the treatment of veterinary cancer patients. NEO212's low toxicity, high therapeutic efficacy in canine lymphoma and leukaemia cells in vitro and in vivo, and ability to be administered orally are all significant advantages compared with current standard of care in the veterinary oncology arsenal against these types of cancers.

## Conflicts of Interest

Thomas C. Chen is the founder, officer, and shareholder of NeOnc Technologies Inc. The other authors declare no conflicts of interest.

## Supporting information


**Data S1.** Supporting Information.

## Data Availability

All data are contained within this article. Further inquiries may be directed at the corresponding author.
