# Supplementary material for: Potent Therapeutic Activity of NEO212 in Preclinical Models of Human and Canine Leukaemia and Lymphoma
Source: Vet Comp Oncol. 2025 May 16;23(3):412–23. doi: 10.1111/vco.13066 (PMC12378078; doi:10.1111/vco.13066)
Supplement: Supplementary file 1 — Data S1. Supporting Information. [file VCO-23-412-s001.docx]

SUPPLEMENTARY MATERIAL

**Potent therapeutic activity of NEO212 in preclinical models**

**of human and canine leukemia and lymphoma**

**Supplementary Figure 1: Design and chemical structure of NEO212**

NEO212 was created by covalently conjugating perillyl alcohol (orange color) to temozolomide (blue color) via a carbamate bridge (green color).

- Supplementary Details to Section 2. Methods —

**2.1. | Cell Culture Conditions**

The human cells were propagated in RPMI 1640 medium supplemented with 10% heat-inactivated fetal bovine serum (FBS; Corning, Glendale, AZ), 2 mM glutamine, 100 U/mL penicillin, and 0.1 mg/mL streptomycin (all from Gibco/Thermo Fisher Scientific, Waltham, MA). RPMI 1640 was prepared with raw materials from Cellgro/MediaTech (Manassas, VA) and provided by the Cell Culture Core lab of the USC/Norris Comprehensive Cancer Center. The canine cells were propagated in RPMI 1640 with supplements as above, and additionally received 1 mM sodium pyruvate, 1% non-essential amino acids, and 1% vitamin solution, all of which were obtained as Gibco-brand 100x stock solutions from Thermo Fisher Scientific (Waltham, MA). All cells were kept in a humidified incubator at 37°C and a 5% CO_2_ atmosphere.

**2.2 | Immunoblots**

Total cell lysates were prepared by disrupting cells with radio-immunoprecipitation assay (RIPA) buffer supplemented with 1 mM phenylmethylsulfonyl fluoride (PMSF) and Halt Protease and Phosphatase Inhibitor Cocktail (all from Thermo Fisher Scientific). Protein concentrations were determined using the Pierce BCA protein assay reagent from Thermo Fisher Scientific. Twenty-five μg of total cell lysate from each sample was mixed with an equal volume of 2x Laemmli loading buffer, boiled for 5 minutes, and separated using denaturing polyacrylamide gel electrophoresis (PAGE) with 12% Mini-Protean TGX pre-cast gels from BioRad (Hercules, CA). This was run in a Mini-Protean Tetra Cell system from BioRad for 35 minutes at 200 volts. The electrophoresis buffer used was Tris/glycine/SDS buffer from BioRad (catalog #1610732). After completion of PAGE, the gel cassette was disassembled and the gel incubated in Transfer Buffer (Tris/glycine buffer [BioRad, #1610734] supplemented with 30% methanol) for 10 minutes at room temperature. The separated proteins were then subjected to semi-dry transfer to a Prometheus polyvinyl-pyrrolidone (PVDF) blotting membrane (Genesee Scientific, Morrisville, NC), using a Trans-Blot SD Semi-Dry Transfer Cell from BioRad. The transfer was performed at 15 volts for 30 minutes, using the Transfer Buffer. After completion of the transfer, the membrane was washed 3 times in deionized water for 1 minute each time, then stained with Ponceau S red solution (Thermo Fisher Scientific) for 15 minutes at room temperature. Destaining was done with two washes in deionized water for 5 minutes each, and a photograph of the membrane was taken.

To block non-specific antibody binding, the membrane was incubated for 1 hour at room temperature in a blocking buffer consisting of 5% bovine serum albumin (BSA) in tris-buffered saline (TBS, BioRad cat. #1706435) with 0.1% Tween20 added [TBST]. The BSA was DNAse-free and protease-free (catalog #BP9706100, Thermo Fisher Scientific). The blocking solution was removed and the membrane was incubated with a primary antibody diluted in fresh blocking solution overnight at 4˚C. The next morning, the primary antibody in blocking solution was removed from the membrane and stored at 4˚C for use on other membranes (re-use was done up to three times). The membrane was washed in TBST for 3 times at 5 mins each time. Thereafter, the membrane was incubated for 1 hour at room temperature with horseradish peroxidase-antibody conjugates (i.e., secondary antibodies) diluted in blocking buffer. This step was followed by three wash steps as above. Residual wash buffer was drained from the membrane and the wet membrane was subjected to the development and detection steps, using SuperSignal West Pico PLUS Chemiluminescent Substrate (Thermo Fisher Scientific). After adding the substrate to the membrane, the signal was detected with the iBright CL1500 Imaging System (Thermo Fisher Scientific) and digital images of the exposure were recorded and used for the preparation of figures.

After signal detection and recording, the membrane was processed for re-probing with a different primary antibody. The membrane was incubated for 15 minutes at room temperature in Restore PLUS Western Blot Stripping Buffer (Thermo Fisher Scientific), which removed the previous primary and secondary antibodies from the membrane. Then, the membrane was again incubated with BSA-containing blocking buffer, followed by the steps outlined above, and incubated with a different primary antibody, secondary antibody, etc.

The following primary antibodies were used. For the detection of cleaved caspase 7: polyclonal rabbit antibody, catalog #9491 from Cell Signaling Technology (Danvers, MA), used at a dilution of 1:1,000. For cleaved caspase 3: monoclonal mouse antibody sc-56053 from Santa Cruz Biotechnology (Dallas, TX), used at 1:200. For PARP-1: rabbit antibody #9542 from Cell Signaling Technology, used at 1:1,000. For phospho-H2AX (Ser 139): mouse monoclonal antibody sc-517348 from Santa Cruz Biotechnology, used at 1:500. For beta-actin: monoclonal antibody sc-47778 from Santa Cruz Biotechnology. Horseradish peroxidase-antibody conjugates (i.e., secondary antibodies) were obtained from Jackson ImmunoResearch Laboratories (West Grove, PA) and used at 1:2,500. All immunoblots were repeated at least once to confirm the results.

**2.3 | MTT Assays**

Methyl-thiazol-tetrazolium (MTT) assays were performed as described earlier.^1^ In short, cells were seeded into 96-well microtiter plates at varying densities from 2 × 10^4^ to 2 × 10^5^ cells/mL and exposed to different concentrations of drugs for different lengths of time. Then, the MTT reaction was performed. The optical density (OD) of each well was measured the next day. In individual experiments, each treatment condition was set up in duplicate or triplicate, and each experiment was repeated several times independently. The graphs in this report show averages derived from different cell densities, taking into account that the chemosensitivity of cells tended to increase as cell densities were lowered (although this had no impact on the qualitative differences between different drug treatments).

**2.4 | Cell Death Analysis by FACS**

Cells were seeded in 10-cm dishes at 2 × 10^5^ cells/mL in regular growth medium and subjected to drug treatment. At the end of the drug incubation period, the cell cultures were transferred to 15-mL conical tubes and gently centrifuged for 5 min. The cell pellets were resuspended in 1 mL of fresh complete medium and further incubated for 5 min at 37˚C with a LIVE/DEAD™ Near-IR Dead Cell probe (Thermo Fisher Scientific) as per manufacturer’s recommendations. This fluorescent probe has peak excitation/emission wavelengths of 633 nm and 775 nm, respectively. After incubation with the probe, the cells were pelleted again and resuspended in ice-cold phosphate-buffered saline (PBS) supplemented with 10% FBS, and kept on ice until FACS analysis. Cell death analysis was performed by flow cytometry on a SORP FACSymphony S6 Cell Sorter (BD Biosciences, Franklin Lakes, NJ), operated by Dialogic Diva 8.5 software (Dialogic, Parsippany, NJ) run on Windows 10 (Microsoft, Redmond, WA). The excitation wavelength was 633 nm, and the emitted light was filtered through a 780/60 filter. Dot plots were generated on which the live and dead cells were gated separately (dead cells stained positive for the Near-IR probe); the dead cells were quantified and percentage numbers are shown in each plot for each treatment condition.

**2.5 | Isolation of RNA and RT-qPCR**

Total RNA from cell lines was isolated using the Quick-RNA Miniprep Kit from Zymo Research (Irvine, CA) according to the manufacturer’s protocol. The RNA concentration was determined using a NanoDrop 2000/2000c Spectrophotometer (Thermo Fisher Scientific). Quantitative RT-PCR was performed using the iTaq Universal SYBR Green One-Step Kit (Bio-Rad, Hercules, CA). PCR reactions were run on an Applied Biosystem 7500 Fast Real-Time PCR System (Thermo Fisher Scientific). All PCR primers were obtained from Integrated DNA Technologies (IDT; Coralville, IA). We used canine-specific primer pair sequences derived from the published literature, as follows: for MGMT and ribosomal protein L32 (RPL32), see Kambayashi et al.;^2^ for beta-2 microglobulin (B2M), see Brinkhof et al..^3^ RPL32 and B2M represent previously validated housekeeping transcripts^3^ that we used in separate, triplicate reactions as the internal controls. The relative expression level of MGMT RNA was calculated using the ∆Cq method with either B2M or RPL32, respectively, as the internal reference. In both instances, comparable results were obtained.

**2.6 | Tumor Cell Implantation and Tumor Take**

For tumor cell implantation, 5-10 × 10^4^ tumor cells (depending on cell type) in a volume of 50 µL phosphate-buffered saline (PBS) were injected intravenously (*via* tail vein) or into the peritoneum. For the canine CLBL1 cell line, we generated luciferase-labeled cells and monitored tumor development in a few representative mice after implantation, in order to confirm effective tumor take and distribution; however, we did not systematically image all cohorts of mice during the course of the experiments. Nonetheless, it was noted that moribund mice displayed symptoms that were consistent with hematological tumor growth.

For human hematological tumors, we investigated blood and spleen samples from some of the euthanized mice for the presence of human cells; this was possible due to the availability of an extremely sensitive qPCR assay that can detect human Alu sequences in the background of mouse cells.^4^ This approach showed us that, in the case of human tumors, representative untreated mice had human tumor cells circulating in their blood (qPCR positive), whereas human tumor cells could not be detected (qPCR negative) in mice treated with NEO212 and euthanized in a healthy state at Day 200 at the end of the experiment. Regrettably, a similar Alu-based assay is not available for canine cells.

**2.7 | Drug Treatment Schedule of Mice**

Between 5 to 12 days after tumor cell implantation, drug treatment was initiated. A variable initiation time point was used to demonstrate the robustness of the treatment schedule, meaning it did not depend on a very early start to become effective. For CLBL1, HL60, and Raji cells, treatment was started on Day 5 after tumor cell implantation, whereas for CLL1390 and U937 cells, it was Day 12.

The mice received treatment *via* oral gavage, consisting of 25 mg/kg NEO212, 25 mg/kg TMZ, or vehicle only. Treatment was once daily for five consecutive days, which was followed by a treatment holiday of 9 days. The 5-day treatment cycle was repeated once more, for two cycles total. In essence, the schedule was 5 days ON/9 days OFF/5 days ON, without any further treatments beyond that, meaning that all treatments were terminated by Day 24 (CLBL1, HL60, and Raji models) or Day 31 (CLL1390 and U937 models).

The vehicle consisted of 10% DMSO, 45% glycerol, and 45% ethanol (vol/vol/vol). NEO212 and TMZ were first dissolved in DMSO and then diluted in ethanol and glycerol to match the vehicle composition.

**2.8 | Criteria for Euthanasia of Mice**

Animals were closely monitored for behavior and general health, body weight measurements were taken regularly, and overall survival was recorded. Mice were not allowed to expire naturally (i.e., death was not an endpoint); instead, euthanasia was performed based on a set of pre-determined criteria, which had been reviewed and approved by IACUC before the start of the experiments. These criteria included decreased food consumption and weight loss (≥20%), labored breathing, hunched posture, lethargy, reduced grooming, and social isolation. At the physiological spectrum, we looked for signs of lymphadenopathy, splenomegaly, hepatomegaly, petechiae, ecchymosis, and pallor of the extremities, all of which can represent symptoms of hematological malignancies. At these endpoints, affected animals were euthanized by intra-peritoneal injection of an overdose of ketamine (160 mg/kg) and xylazine (20 mg/kg), followed by cervical dislocation.

**REFERENCES**

1. Silva-Hirschberg C, Hartman H, Stack S, et al. Cytotoxic impact of a perillyl alcohol-temozolomide conjugate, NEO212, on cutaneous T-cell lymphoma in vitro. *Ther Adv Med Oncol.* 2019;11:1758835919891567.

2. Kambayashi S, Minami K, Ogawa Y, et al. Expression of O(6)-methylguanine-DNA methyltransferase causes lomustine resistance in canine lymphoma cells. *Can J Vet Res.* 2015;79(3):201-209.

3. Brinkhof B, Spee B, Rothuizen J, Penning LC. Development and evaluation of canine reference genes for accurate quantification of gene expression. *Anal Biochem.* 2006;356(1):36-43.

4. Funakoshi K, Bagheri M, Zhou M, Suzuki R, Abe H, Akashi H. Highly sensitive and specific Alu-based quantification of human cells among rodent cells. *Sci Rep.* 2017;7(1):13202.
